# Purifying selection of long dsRNA is the first line of defense against false activation of innate immunity

**DOI:** 10.1186/s13059-020-1937-3

**Published:** 2020-02-07

**Authors:** Michal Barak, Hagit T. Porath, Gilad Finkelstein, Binyamin A. Knisbacher, Ilana Buchumenski, Shalom Hillel Roth, Erez Y. Levanon, Eli Eisenberg

**Affiliations:** 1 The Mina and Everard Goodman Faculty of Life Sciences, Bar-Ilan University, Ramat-Gan, 52900 Israel; 2 Raymond and Beverly Sackler School of Physics and Astronomy and Sagol School of Neuroscience, Tel Aviv University, Tel Aviv, 69978 Israel

## Abstract

**Background:**

Mobile elements comprise a large fraction of metazoan genomes. Accumulation of mobile elements is bound to produce multiple putative double-stranded RNA (dsRNA) structures within the transcriptome. These endogenous dsRNA structures resemble viral RNA and may trigger false activation of the innate immune response, leading to severe damage to the host cell. Adenosine to inosine (A-to-I) RNA editing is a common post-transcriptional modification, abundant within repetitive elements of all metazoans. It was recently shown that a key function of A-to-I RNA editing by ADAR1 is to suppress the immunogenic response by endogenous dsRNAs.

**Results:**

Here, we analyze the transcriptomes of dozens of species across the Metazoa and identify a strong genomic selection against endogenous dsRNAs, resulting in their purification from the canonical transcriptome. This purifying selection is especially strong for long and nearly perfect dsRNAs. These are almost absent from mRNAs, but not pre-mRNAs, supporting the notion of selection due to cytoplasmic processes. The few long and nearly perfect structures found in human transcripts are weakly expressed and often heavily edited.

**Conclusion:**

Purifying selection of long dsRNA is an important defense mechanism against false activation of innate immunity. This newly identified principle governs the integration of mobile elements into the genome, a major driving force of genome evolution. Furthermore, we find that most ADAR1 activity is not required to prevent an immune response to endogenous dsRNAs. The critical targets of ADAR1 editing are, likely, to be found mostly in non-canonical transcripts.

## Introduction

Most invading viruses give rise to long double-stranded RNAs (dsRNAs) in the cytoplasm of the host cells [1]. These structures are identified by sensor proteins such as MDA5 and trigger the production of type I interferons as part of recruiting the innate immunity system [2, 3]. However, large numbers of endogenous dsRNAs are likely to appear in normal eukaryotic cells as well [4], mainly due to the abundance of mobile elements in the genome—transcripts harboring nearby inverted copies of the same repeat fold to create an endogenous dsRNA structure [5]. Therefore, distinguishing between self and non-self dsRNAs is critical for proper innate immune activity.

It was pointed out recently that the main function of the essential ADAR1 enzyme, conferring adenosine to inosine (A-to-I) RNA editing, is to prevent activation of cytosolic immune response [6–10]. A-to-I editing, mostly carried out by the constitutive ADAR1p110 variant, introduces mismatches to the endogenous dsRNAs while still in the nucleus [11, 12], so that the edited endogenous transcripts are no longer recognized by dsRNA sensors in the cytoplasm. As mobile element activity goes on, more and more putative dsRNAs accumulate in the transcriptome, and the burden on ADAR1, constitutively marking all of these targets to prevent false activation of innate immunity, becomes increasingly heavier. Therefore, one may hypothesize that even in the presence of ADAR1 editing, endogenous dsRNAs have a detrimental effect and should be selected against. Such selection would then have an impact on the proliferation of repetitive elements in the DNA, a major driving force for genome evolution [13].

Furthermore, editing levels are usually quite low within paired inverted Alu repeats, the archetypal examples of human endogenous dsRNAs [14–17]. Although many adenosines are editable in each Alu repeat, most of these are converted into inosine only in < 1% of the transcripts [18]. Accordingly, the average number of inosines per double-stranded region in an RNA molecule is often lower than one, at least for the less-edited tissues, and the majority of individual molecules are not edited at all [18]. Thus, it seems that editing cannot fully protect from false activation of innate immunity by these endogenous dsRNAs, or else that the typical editing target does not pose a risk of such false activation, edited or unedited.

Here, we study the repertoire of putative dsRNA structures in mRNA and pre-mRNA molecules. We analyze the transcriptomes of 49 organisms, from yeast to human, and find that dsRNAs in mRNA molecules are strongly depleted from the genome. This demonstrates a strong selective force that controls the integration of repetitive elements into genes in order to minimize the number of endogenous dsRNAs. Notably, in pre-mRNA molecules (including the introns), such depletion is much weaker. This indicates that the main driving forces of this selection process are cytoplasmic, presumably the need to avoid activation of innate immunity sensors by endogenous RNA. Moreover, long and nearly perfect RNA duplexes, reported to be the prime targets of MDA5 [19, 20], are strongly depleted even in pre-mRNA or expressed at a low level leading to a weaker selective pressure. Finally, we find that some of the few long and nearly perfect dsRNAs that are not purified from the human genome undergo heavy RNA editing. Such loci may be critical ADAR1 targets.

## Results

In order to explore the extent to which mature RNA molecules and pre-mRNA molecules form long and stable dsRNA structures, we employ a cross-species whole transcriptome approach, studying the full transcriptome of 49 organisms included in the Ensembl database [21], from yeast to human (Additional file 1: Table S1). Altogether, we analyzed pre-mRNA (genomic sequence from the beginning of the first exon to the end of the last exon, including introns) and the much shorter mature RNA sequences for 724,071 different genes (14,777 per organism, on average), covering 21.7 Gbp.

**Fig. 1:**
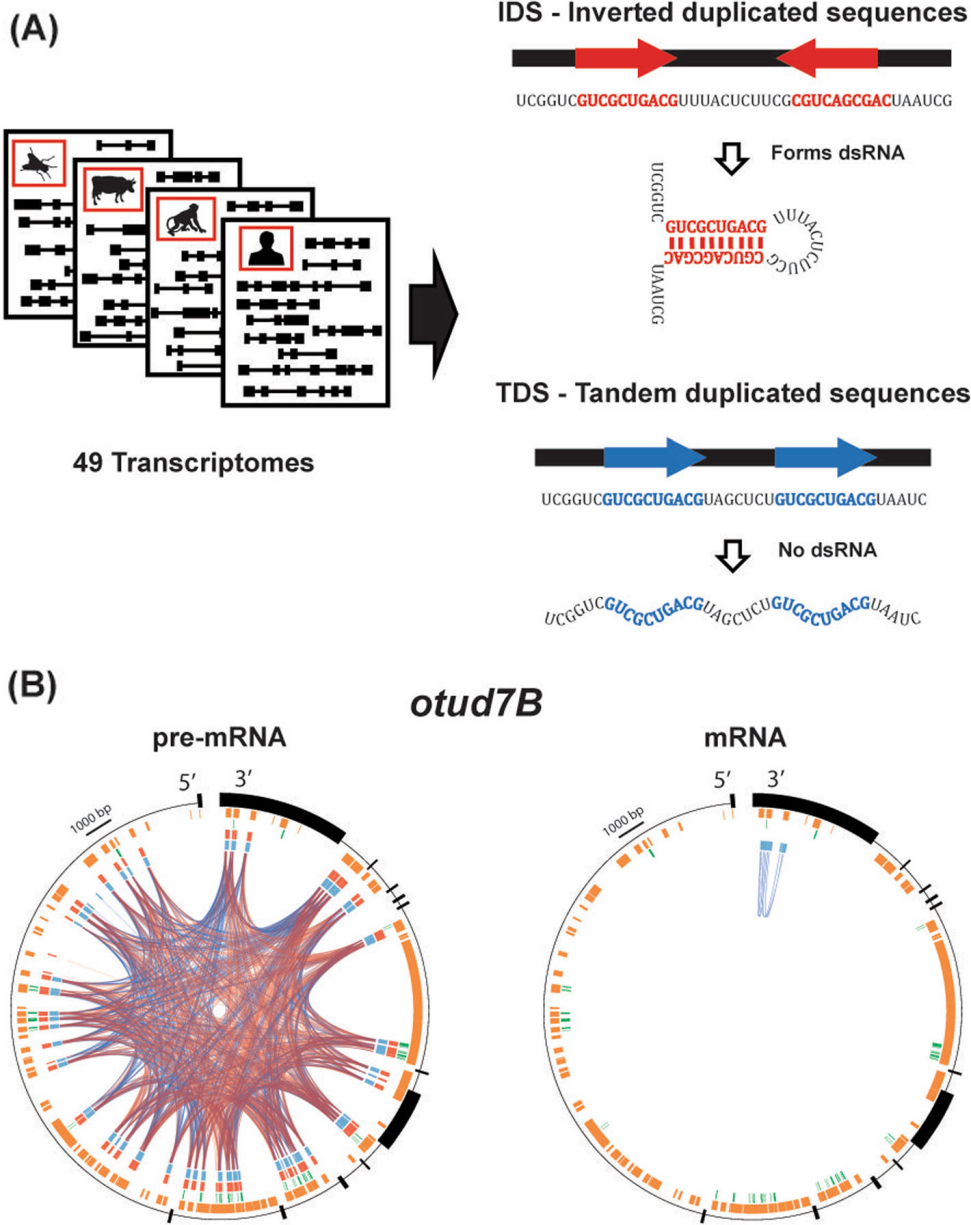
Detection of putative long dsRNA structure across transcriptomes. **a** Transcriptomes of 49 organisms, from yeast to human, were analyzed. Using BLAST, we searched for long highly similar sequences within the same mRNA and pre-mRNA sequence. Reversely oriented sequences (red) are likely to pair and form a long intra-molecular dsRNA structure. As a natural control, we use same-strand tandem duplicated sequences (blue), which are not relevant to the secondary structure. **b** For example, looking at the pre-mRNA of the human *otud7b* gene, multiple same-strand (blue) and inverted (red) matches are found, most of these pair together repetitive elements (orange) within introns (thin black line). In contrast, the mRNA molecule shows very few hits, all of them pair tandem sequences within repetitive elements in the 3′ UTR. Green bars represent A-to-I editing events, all of which are located in regions that have an inverted sequence match in the pre-mRNA

Despite important recent technological advancements, predicting the detailed structure of RNA molecules, including structural motifs, protein-binding regions, and RNA-RNA interactions, is yet a major challenge. Numerous computational tools are available for predicting RNA secondary structures, but their reliability in predicting full-length molecule structure is limited [22]. Thus, full understanding of the RNA folds requires intricate experimental approaches, currently inapplicable at large scale [23]. However, here, we are not interested in the full and accurate structure, but rather focus on long dsRNA stems, > 40 bp long. These substructures are easily detected by standard sequence alignment tools, e.g., BLAST [24]—if a long region of a molecule is highly similar to the reverse complement of another region in the same molecule, these two regions are likely to pair together and form a long and stable dsRNA. We thus use BLAST to align each of the mRNA and the pre-mRNA sequences to itself, and count the number of reversely oriented duplicated sequences (inverted duplicated sequences, IDS) as well as the total number of nucleotides involved. BLAST is not a perfect predictor of dsRNA stems, as it does not take into account the different pairing energies of A:T and G:C pairs and ignores the binding energy of G:U pairs. However, the size of the database we searched prevents using the much slower RNA folding algorithms, and for long and nearly perfect stems, BLAST provides a reasonable approximation. As a natural control, we look at the number of same-strand hits, showing similar sequence identity for regions on the same strand (tandem duplicated sequences, TDS), which are not relevant to the secondary double-stranded structure (Fig. 1).

**Fig. 2:**
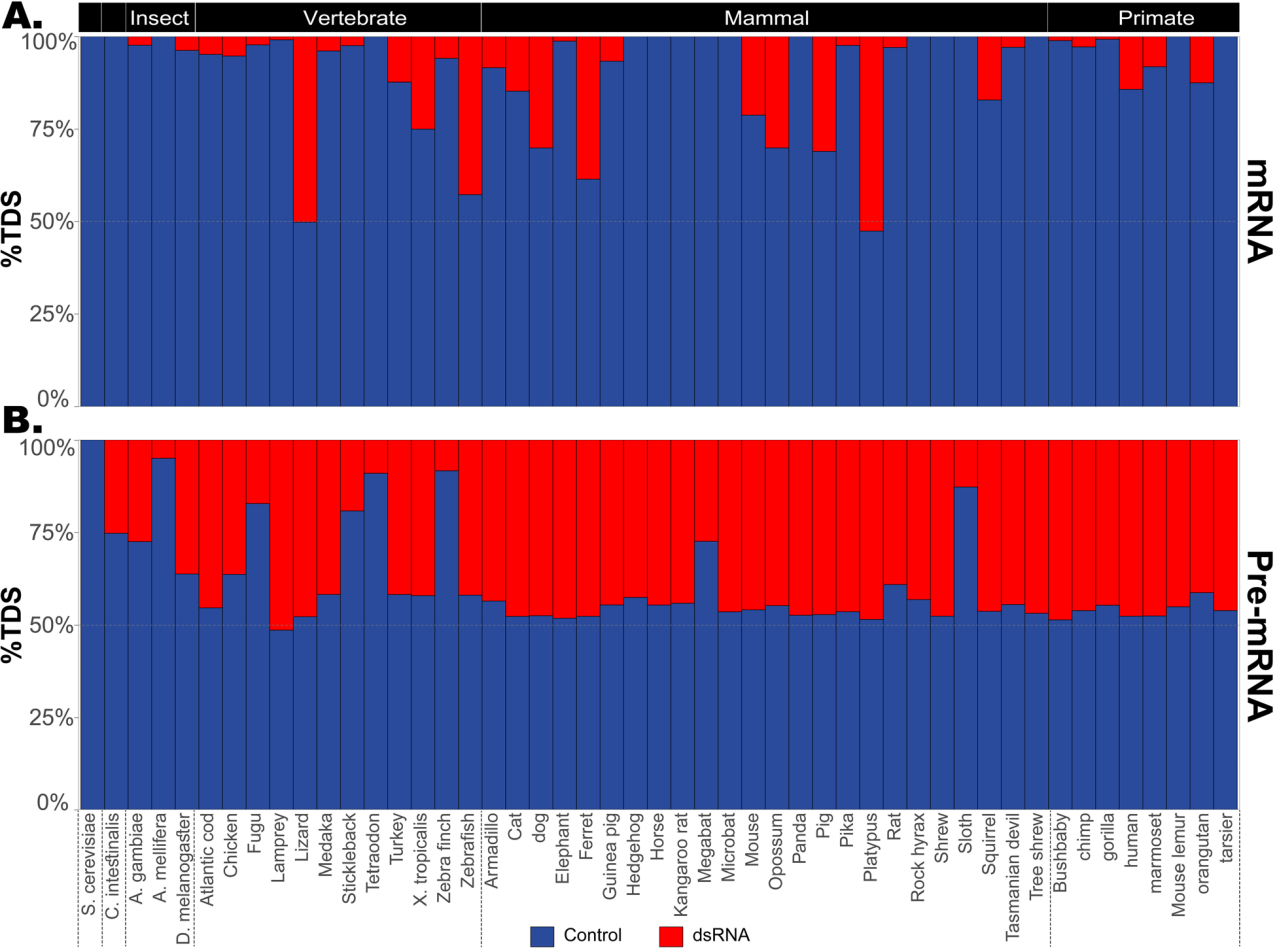
Putative dsRNAs are depleted in mRNA across organisms. Comparison of inverted duplicated sequences (potentially folding into dsRNA; red) to tandem duplicated sequences (control; blue) across a wide range of organisms. For each organism, we present the relative abundance of each of the two types of alignments. **a** In the mRNA molecules, the potential for dsRNA formation is strongly depleted in most organisms. **b** In contrast, pre-mRNA molecules exhibit, for most organisms, a number of potential dsRNA regions similar to that of the control

One immediately notices a global depletion of IDSs in mRNA molecules, compared to the control TDSs (see Fig. 2). Looking at all organisms combined, one finds only 6525 IDSs in mRNAs covering 786 kbp, compared with 42,946 TDSs covering 3.01 Mbp (*p* < 1e−100; see Additional file 1: Table S2). This depletion indicates a strong selection against long and stable duplexes in the mRNA, consistent across most organisms. Furthermore, the selection against dsRNAs is much weaker in the pre-mRNA, suggesting it is driven by cytoplasmic processes (57.5M IDSs covering 1.26 Gbp, compared with 61.3M TDSs covering 1.51 Gbp; *p* < 1e−100). Limiting the gap between the 2 duplicated sequences to 2000 bp does not change the results, qualitatively (Additional file 2: Figure S1). This is consistent with the notion of avoidance of innate immunity activation by endogenous RNA, as viral dsRNA sensors are active in the cytoplasm [25]. Note, however, that stronger depletion of dsRNAs in pre-mRNAs is observed in a few species. For example, yeast introns are uncommon [26], and thus, the pre-mRNA is almost identical to the mRNA. Similarly, while honeybees do have an ADAR enzyme [27], very few retroelements have been identified in the honeybee genome [28], which could account for the lower number of dsRNAs in introns.

**Fig. 3:**
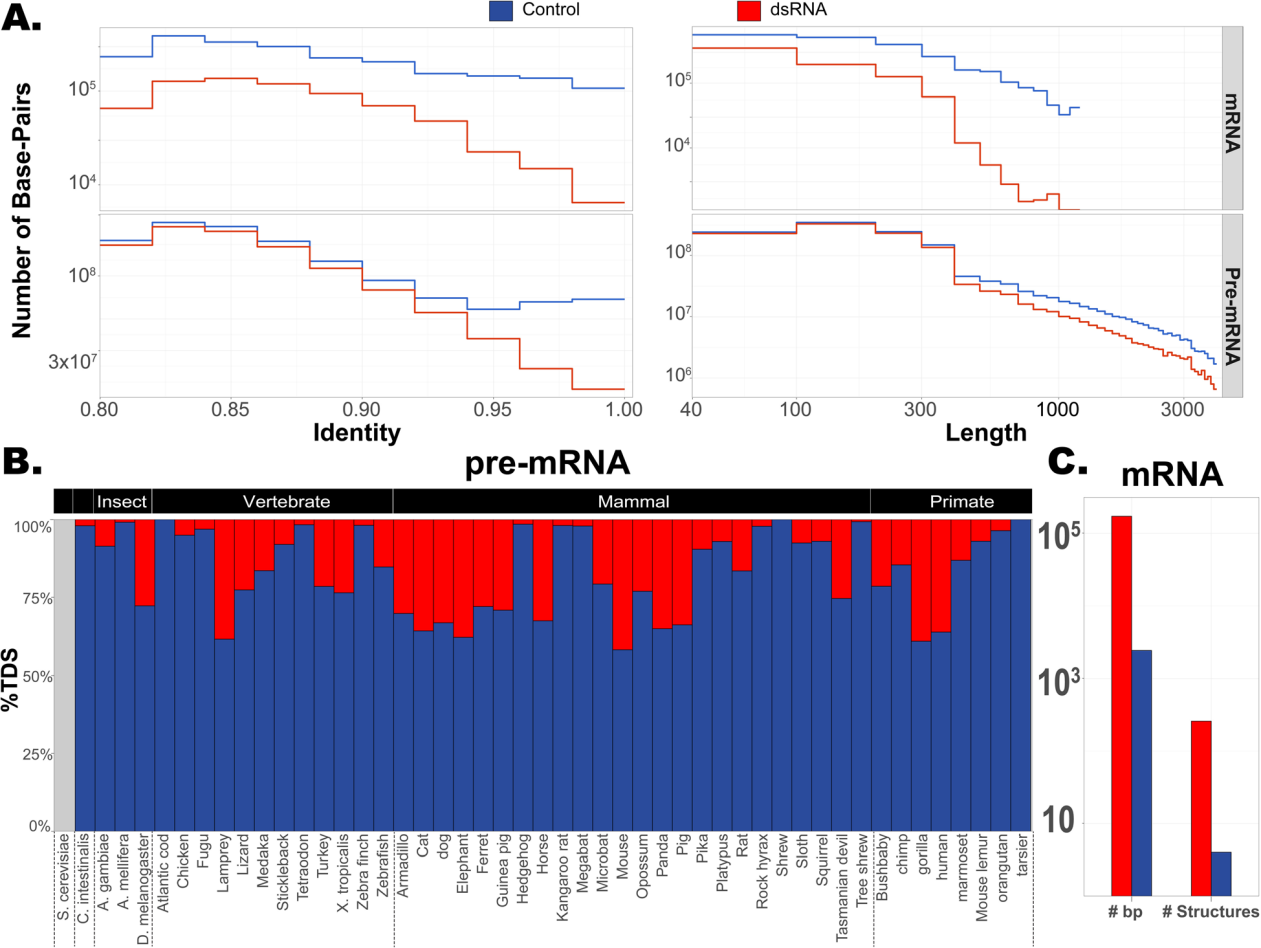
Long and nearly perfect duplexes are extremely rare. **a** Number of genomic nucleotides within putative dsRNAs (IDS) and control (TDS) regions, summed over all organisms, as a function of the region length (right) and the similarity (left), for both mRNA (top) and pre-mRNA (bottom). Note the logarithmic scale. For regions longer than 300 bp and of very high identity, the depletion of putative dsRNAs becomes more pronounced, even in pre-mRNAs. **b** Comparison of long (> 300), nearly perfect (> 96%), inverted duplicated sequences (potentially folding into dsRNA; red) to tandem duplicated sequences (control; blue) across a wide range of organisms, for pre-mRNA molecules. In most organisms, these putative dsRNA structures are completely depleted. Gray indicates no data (zero IDS and zero TDS). **c** In mRNA, there are only 4 long and nearly perfect structures in all organisms combined (2419 bps), compared with 258 control TDSs (172,326 bps)

Long and nearly perfect duplexes are the prime candidates to provoke an innate immune response, as they resemble viral dsRNAs [25, 29]. Consistently, we observe a stronger genomic depletion for longer and highly base-paired structures (Fig. 3 and Additional file 2: Figure S2). There are only 246 almost perfectly base-paired IDSs (putative helices with > 96% identity), compared with 3436 almost perfect TDSs (32,245 bp vs. 425,753 bp) in all organisms (*p* < 1e−100). Moreover, depletion of almost perfect hits is noticeable even for pre-mRNAs (1.07M TDSs vs. 0.78M IDSs (*p* < 1e−100); 205 Mbp vs. 81 Mbp, respectively; Additional file 2: Figure S2 and Additional file 1: Table S3). Similarly, strong depletion is observed for long (> 300 bp) IDSs in mRNAs, with only 195 structures covering 91,310 bp, compared with 3915 TDSs covering 1.41 Mbp (*p* < 1e−100). In pre-mRNA molecules, one finds only 1.57M long IDSs compared with 2.31M long TDSs (*p* < 1e−100; 401 Mbp vs. 608 Mbp, respectively) (Additional file 2: Figure S2 and Additional file 1: Table S4). Finally, IDSs that are both long and almost perfectly matching are extremely rare, only 4 such examples are present in mRNA sequences of all organisms examined (manual inspection suggests these are unreliable), compared with 258 such TDSs (*p* = 2.6e−71; 54,479 vs. 144,989 structures in pre-mRNA) (Fig. 3 and Additional file 1: Table S5). We conclude that long and nearly perfect dsRNA structures are almost nonexistent in mature RNAs and selected against even if they are present only in the pre-mRNA molecules. A possible explanation for the depletion in pre-mRNA is that regions annotated as introns might actually be mis-annotated exons (constitutive or alternative), especially in less-explored transcriptomes. In addition, there might be a selective pressure even against intronic dsRNAs due to the occasional intron retention as a result of imperfect splicing. Although rare, dsRNAs present in these aberrantly spliced transcripts may trigger the immune system and therefore are selected against.

**Fig. 4:**
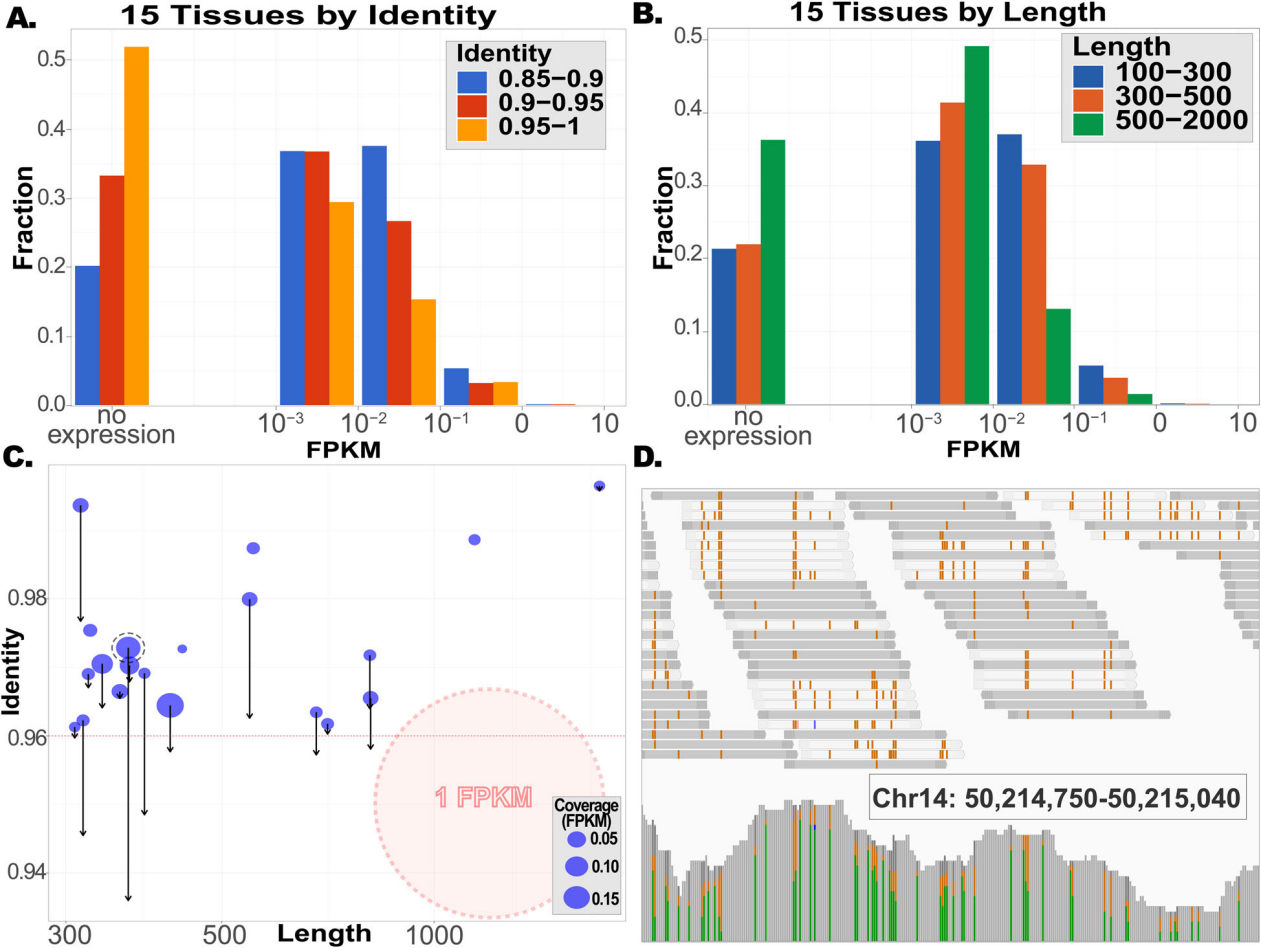
Expression and editing of long and nearly perfect duplexes. Depletion of long and nearly perfect dsRNAs from the human transcriptome is more pronounced for regions that are expressed more strongly. In this figure, expression was calculated based on a pool of 30 GTEx samples, from 15 different tissues (Additional file 1: Table S6). **a** Distribution of dsRNA tightness (%identity) for several expression levels. Many of these regions are not expressed at all, and the ones that are (very weakly) expressed show a reduced fraction of nearly perfect (> 95%) structures. **b** Similarly, long structures are depleted in the expressed regions, compared with the ones that show no expression. **c** Twenty long and nearly perfect human structures were expressed at a level exceeding FPKM = 0.01 (roughly, 0.002–0.02 RNA molecules per cell [31]). Seven of these (including the two structures expressed at levels exceeding FPKM = 0.1) are indeed edited appreciably (black arrows), possibly bringing the edited structure below 96% identity. The single point marked by a dashed line corresponds to the data presented in **d**. **d** Actual editing pattern for one of the unwound structures. A putative dsRNA structure, located within an intron of *klhdc1* (chr14:50213276-50215083), is expressed in our pool at a level of FPKM = 0.13. These transcripts are heavily edited, with an estimated number of 14 inosines per transcript, on average. Top: reads mapped to 1 of the 2 arms of the structure (see region coordinates noted in the panel). Data accumulates reads from the 30 pooled samples. Editing events (A-to-G mismatches) show up in brown. Bottom: pile-up of the reads coverage, 59 different editing sites are observed in this 291-bp-long region. They are marked by green and brown bars (standing for A and G fractions, respectively)

To further support the idea that depletion of IDSs within transcripts is related to the potential risk of endogenous dsRNAs, we study the expression level of all IDSs identified within RefSeq human transcripts in a pool of 30 RNA-seq GTEx [30] samples, originating from 15 different tissues (Additional file 1: Table S6). We find that the expression of IDSs negatively correlates with their length and identity (Fig. 4a, b), as expected if the driving selective disadvantage relates to the expressed RNA molecules. To exclude the possibility of reverse-transcription artifacts related to the secondary structures, we verified that the regions that show no expression in mRNA-seq data are actually well-covered in total RNA datasets, suggesting that the reverse transcription does allow their amplification (Additional file 2: Figure S3). Note that expression here refers to that of the IDS region, which could be much lower than the expression level of the hosting gene, as the IDS is mostly due to intronic sequences.

The above results suggest that the main suppressor of innate immune response that may be triggered by endogenous double-stranded RNAs is a tight purifying genomic selection. However, the balance between the continuous introduction of new putative dsRNAs, mainly due to the proliferation of repetitive elements, and the pruning of dangerous dsRNAs by purifying selection may lead to a residual number of potentially dangerous structures. In fact, we do see that most dsRNA structures reside in repetitive sequences (counting unique dsRNA genomic nucleotides overlapping known repeats: 531/786 kbp in mRNAs and 707/1260 Mbp in pre-mRNAs, for organisms with repetitive elements annotation; Additional file 1: Table S7). Furthermore, the depletion of IDSs in mRNA (but not pre-mRNA) is stronger for dsRNAs not associated with repetitive elements (Additional file 2: Figure S4). These observations are consistent with the view that dsRNAs associated with repetitive elements are being continuously added to the genome, and the purifying selection against them is still ongoing, maintaining the number of long and nearly perfect self-dsRNAs under control.

It was suggested recently that editing by ADAR1 plays an important role in preventing the activation of cytosolic response [7–10]. Using the abovementioned pool of GTEx tissues, we studied the editing levels in all 197 human IDSs within RefSeq transcripts that are both longer than 300 bp and with identity above 96% (Additional file 1: Table S8). Of these, in only 20 (10%), both arms of the IDSs are expressed at a level exceeding FPKM = 0.01 (to give perspective, FPKM = 1 corresponds, roughly [31], to 0.2–2 RNA molecules per cell). Seven of these are indeed edited strongly enough to bring the edited structure below the 96% identity cutoff (Fig. 4c). Only 2 IDSs are expressed at levels exceeding FPKM = 0.1, and both become much less base-paired by editing (one of these is presented in detail in Fig. 4d). Looking at another pool of 30 samples from the weakly edited muscle tissue (Additional file 1: Table S6), only 12 IDSs (6%) pass the FPKM = 0.01 cutoff, and in 5 of them, editing brings the identity below 96%. Not a single IDS is expressed stronger than FPKM = 0.1 in this pool (Additional file 1: Table S8).

We thus find that purifying genomic selection is the main contributor to protection against false activation of a cytosolic response to canonical endogenous transcripts appearing in a reference transcriptome, such as RefSeq. To the extent ADAR1 editing has any role in this protection, it is limited to a handful of putative dsRNA structures. Clearly, the vast majority of ADAR1 editing activity is irrelevant for this protective role. Furthermore, many of the essential targets of ADAR1 reside, likely, out of canonical transcripts.

## Discussion

Most viral infections lead to long viral dsRNAs in the cytoplasm [1]. These are recognized by the innate immunity sensors [2] and trigger a response involving activation of hundreds of genes, mainly through interferon type I pathways [3]. Other dsRNA-based antiviral defense mechanisms are observed in organisms lacking the interferon pathway, such as the siRNA pathway in insects [32]. This response to viral infections comes at a price of severe damage to the hosting cell and its surroundings and may even affect the entire organism. Therefore, it is crucial to prevent the misidentification of endogenous dsRNAs as a viral infection that would trigger the above response unnecessarily [33].

Throughout the course of evolution, organisms are bound to accumulate more and more new dsRNA structures. Most endogenous long and stable dsRNA structures are due to intra-molecular folding, rather than binding of two (possibly anti-sense) transcripts [34]. Genomes are continuously bombarded by mobile elements that are often integrated into the genomic sequence. In many cases, the appearance of an active new element results in a dramatic accumulation of numerous nearly identical copies of the same element over a short period of time. These provide a natural source of new dsRNA structures—two reversely oriented copies of the same mobile elements introduced into a transcribed region of the DNA will be transcribed into the RNA molecule and form a long and nearly perfect dsRNA structure [5]. The results presented here show how this influx of novel putative dsRNAs into the transcriptome is encountered by a global purifying selection.

Purifying selection is a major driving force of evolution, weeding out of the genome harmful alleles. Here, we demonstrated a general evolutionary principle that provides a fundamental layer of defense against false triggering of the viral infection response: Endogenous dsRNA-forming sequences that pose a risk of self-intolerance leading to cell death are either rooted out of the genome or silenced transcriptionally.

What is then the critical role of ADAR1 editing? As mobile element activity goes on, more and more repeats integrate into the transcribed part of the genome, and putative dsRNAs accumulate in the transcriptome [35]. Some of these newly added sequences may pose a risk of undesired immune response. In the long run, these structures are likely to be eliminated from the genome through purifying selection, possibly even before they are fixated in the whole population. However, the balance between these two counteracting processes is bound to lead to a residual number of somatic or polymorphic potentially dangerous transcripts. Alternatively, such offending structures may appear in lowly expressed alternatively spliced exons or rarely expressed long 3′ UTRs. We hypothesize that handling these few targets is the raison d’etre of ADAR1 editing activity.

Strong depletion is observed for long and nearly perfect dsRNAs, consistent with previous studies of MDA5/MAVS specificity. However, short (< 300 bp) and imperfect (identity < 96%) dsRNAs are also purified from mRNAs. Why would these be depleted? Possibly, even shorter and imperfect RNA duplexes may be recognized by dsRNA sensors to some extent. Alternatively, an overload of ADAR1-binding transcripts might have a detrimental effect on ADAR1 protection, even if these transcripts themselves do not pose a risk. In addition, endogenous dsRNAs might have additional detrimental effects on RNA processing and translation [36–39].

Integration of mobile elements is one of the major mechanisms for genomic innovations [40]. To date, not much is known of the rules governing these integration events. One of the few observations regarding mobile element fixation relates to a bias for same-strand orientation of neighboring elements due to recombination, specifically demonstrated for the primate-specific Alu element [41–43]. However, the depletion of dsRNAs found here is much stronger and specific to the expressed mRNA sequences. It even varies between mature RNAs and pre-mRNA regions. Thus, the selective force demonstrated here is essentially different in that it is governed by the RNA, and not the DNA, structure. Specifically, it is determined by distances on the mature RNA molecule, irrespective of the physical distance along the chromosome. The selective effect described here is therefore a new important rule shaping genome structure and evolution.

Finally, we would like to note that most of ADAR1 editing is not at all required for preventing the innate immune response. For example, most editing events in human exons occur due to exon-intron pairing. These exonic Alu elements do not have any complementary Alu sequence in the hosting mRNA sequence after splicing and thus do not pose any threat of dsRNA formation at the mRNA level. Moreover, these are typically edited in about one adenosine per Alu sequence [18], which is usually unlikely to confer major changes in the secondary structure. In addition, the majority of ADAR activity occurs in intron-intron pairs and modifies RNA sequences that are not transported to the cytoplasm at all. Thus, the fraction of ADAR1 activity that takes care of the rare endogenous dsRNA structures in mature RNA that poses a threat of misidentification by the innate immunity system is astonishingly minute. This testifies to the critical importance of handling these rare, recently introduced, dsRNAs that have yet evaded the genomic selection mechanism.

## Methods

### Transcriptome data

Ensemble [21] transcriptomes were downloaded from the UCSC site [44] for all 53 available organisms from different clades. We excluded 3 organisms for which < 200 genes were reported, as well as 1 case where there was an inconsistency between the mRNA and pre-mRNA data (Additional file 1: Table S1). Pre-mRNA transcripts longer than 1 million bp were discarded. Overlapping transcripts were removed, keeping only the longer variant for each gene. Genes with several genomic duplications were included only once. Genes mapped to mitochondria, haplotypes of standard chromosomes, and random or unknown chromosomes of the hg19 assembly were discarded. The organisms in our set are not all equally explored, leading to large variations in the reliability and coverage of the annotated transcriptomes. To partially mitigate this, we kept only protein-coding genes, which are better characterized. These filters resulted in 724,809 genes, 1 transcript per gene, with a total pre-mRNA length of 1.37 Gbp.

### Quantifying the load of putative dsRNAs

For each gene, we used BLAST [24] to look for alignments of the sequence to itself, for both the pre-mRNA and the mRNA sequences, keeping only matches with length > 40 and identity > 70%. BLAST matches involving the two strands (plus/minus hits) are considered putative dsRNAs, while same-strand hits (plus/plus) are used as a control (Fig. 1b).

Often, the same region appears in multiple hits. We thus created a bed file from all reversely oriented or same-strand hits in an organism (within a given range of identity and length), merged the regions using bedtools sort and merge, and summed the number of genomic nucleotides in the merged regions. A nucleotide belonging to several hits was assigned to the longer of them.

### Expression and editing in human tissues

We compiled a pool of 30 GTEx RNA-seq samples originating from 15 different human tissues (2 samples each) and another pool of GTEx muscle samples (representing a lowly edited tissue) (see Additional file 1: Table S6). Duplicated reads were removed using PRINSEQ lite (http://prinseq.sourceforge.net/index.html). RNA-seq data were aligned (as single-end reads) to the genome (hg19) using BWA [45] with default parameters. To account for heavily edited reads, reads that failed to align were re-aligned using the hyper-editing 3-letter approach [46], with default parameters. Some of the studied dsRNA regions are composed of 2 nearly identical regions. Thus, in addition to the uniquely aligned reads, we also included reads that were mapped to both arms of the putative dsRNA region and in an opposite orientation (they were assigned to one of the arms, randomly). Altogether, 1.343 billion (single-end) reads were mapped for the pool of 15 tissues and 1.419 billion for the muscle samples. In both cases, more than 99.9% of these were uniquely mapped by BWA.

Following alignment, we trimmed 5 bases from both ends of the read using trimBam of BamUtil [47]. We then ran samtools mpileup [48] to find the number of reads matching the genomic A and the number of A-to-G mismatches, for each genomic adenosine within putative dsRNA regions. The sum of these 2 is the coverage of the genomic nucleotide, and the ratio of mismatches to coverage is its editing level. For each arm of the putative dsRNA, we define the coverage as the average coverage over all the adenosines within, and the editing index [12, 49] as the ratio of all A-to-G mismatches mapped to the region to the sum over the coverage of all adenosines. FPKM values were calculated based on the total number of reads used and their effective length following trimming. For example, 1.343 billion reads in the 15 tissue pool, with an effective length of 66 bp (after trimming 5 bp from each end of the 76-bp reads), translate to 1 FPKM = 88.64 coverage. We also calculate the average number of inosines per dsRNA structure (index times the number of adenosines). To estimate the contribution of editing to destabilization of the dsRNA structure, we define the residual identity between the arms as the original identity between the genomic sequences minus the ratio of average inosine number to region length (average length of both arms). This assumes that all inosines lead to destabilization, which is approximately correct for the tightly bound structures of interest here. For this analysis, we considered only dsRNA regions where both arms reside within the same RefSeq transcript. Overlapping dsRNA regions were discarded for this calculation, retaining only the one with the highest identity.

In order to exclude the possibility of no coverage due to technical reverse-transcription problems, we analyzed the expression level in the same regions for a dataset of total RNA-seq from 22 healthy human mammary tissues (GSE103001 [50]). We used the expression of a list of housekeeping exons [51] to normalize the expression FPKM values across mRNA and total RNA samples.

### Repetitive elements

To calculate the fraction of paired regions belonging to genomic repeats, we used RepeatMasker annotation as downloaded taken from UCSC (when available). Tetraodon was excluded from this analysis, as there was no RepeatMasker annotation for this organism.

### Statistics

To test for significance of the disparity between numbers of IDS and TDS, we look at the observed fraction of IDSs among all duplicated sequences found, i.e., #IDS/(#IDS+#TDS), and use a single proportion test to reject the null hypothesis that IDS and TDS are equally probable (i.e., the fraction is 0.5 for the parent distribution).

## Supplementary information

**Additional file 1:** Supplementary tables.

**Additional file 2:** Supplementary figures.

**Additional file 3:** Review history.

## Data Availability

All sequencing data used in this study are publicly available. RNA-seq reads for this analysis were obtained from GTEx (see “Methods” section and Additional file 1: Table S6 for the list of RNA-seq files used). Ensembl transcriptomes were downloaded from the UCSC genome browser site (see the “Methods” section).
