# Supplementary material for: Purifying selection of long dsRNA is the first line of defense against false activation of innate immunity
Source: Genome Biol. 2020 Feb 7;21:26. doi: 10.1186/s13059-020-1937-3 (PMC7006430; doi:10.1186/s13059-020-1937-3)
Supplement: Supplementary file 2 — Additional file 2: Supplementary figures. [file 13059_2020_1937_MOESM2_ESM.docx]

**Supplementary Figures**


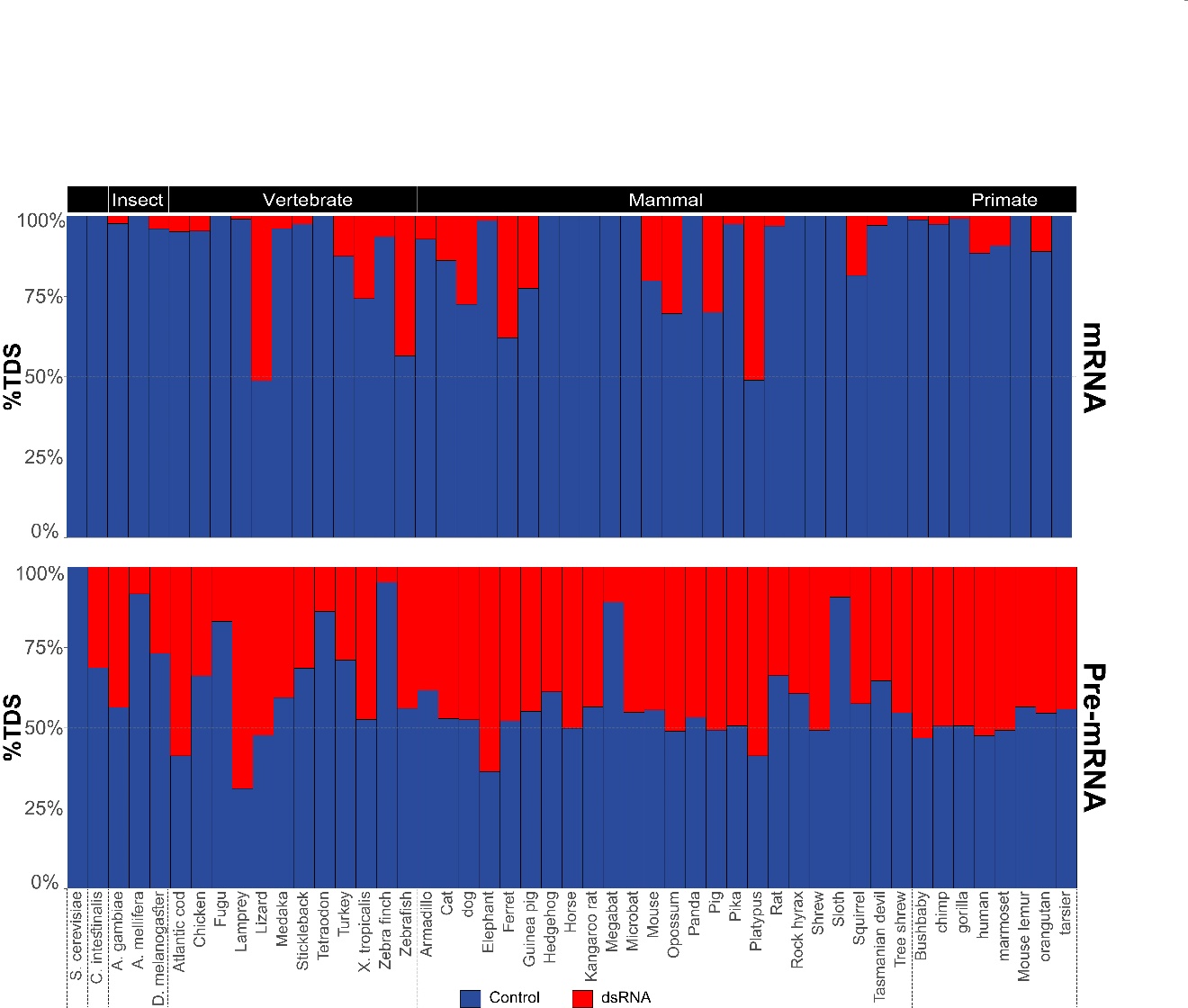
**Figure S1**: **Depletion of dsRNAs from mRNAs, but not pre-mRNAs, for short-gap structures**. Same as Figure 2, for IDSs and TDSs formed by duplicated sequences that are less than 2000bp apart. The qualitative results are the same: IDSs are depleted from mRNAs, but not from pre-mRNAs, suggesting a cytoplasmic explanation.

**
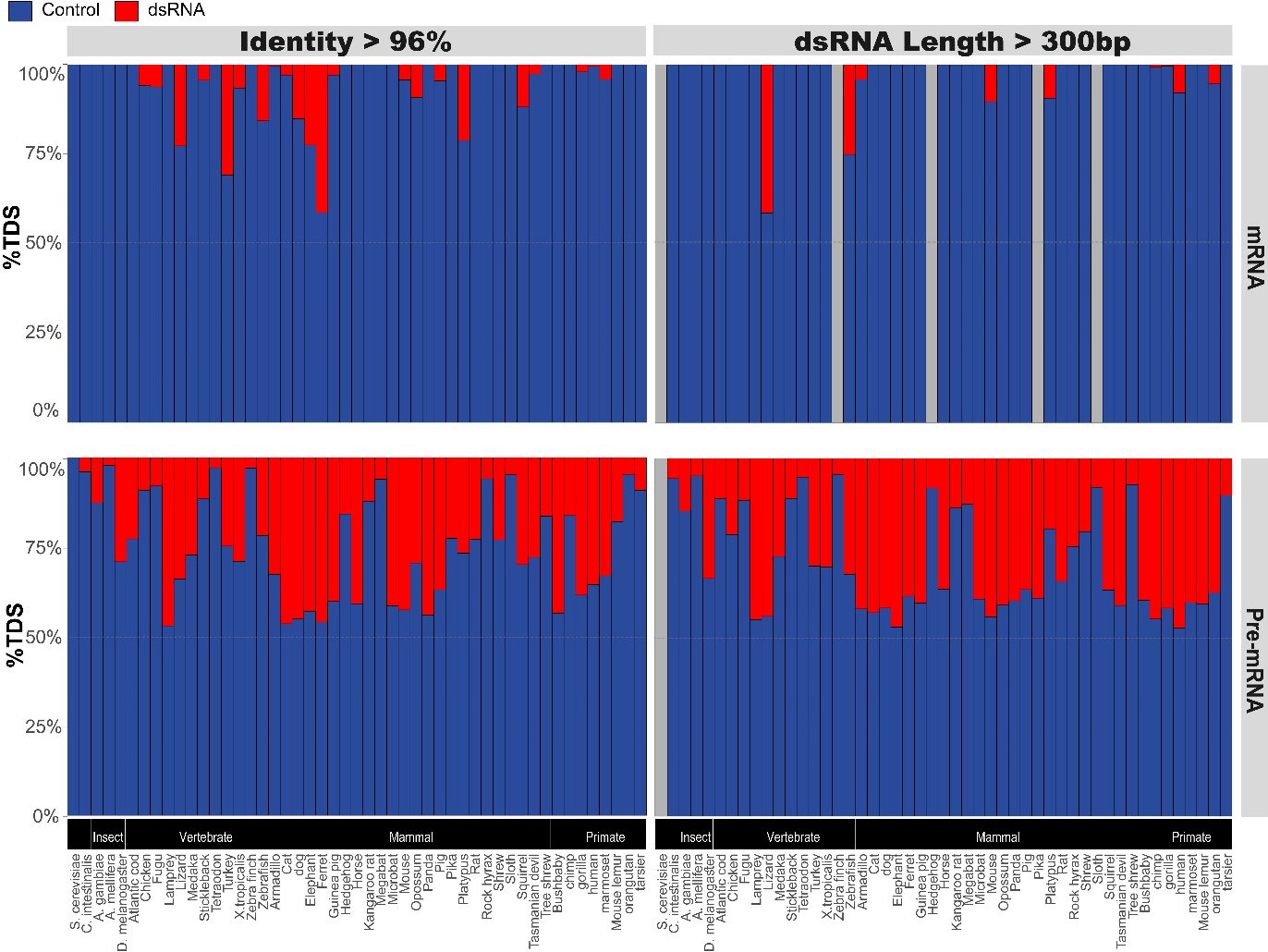
**

**Figure S2**: **Long, nearly perfect, duplexes are extremely rare**. Right: Comparison of long (>300bp) inverted duplicated sequences (potentially folding into dsRNA; red) to tandem duplicated sequences (control; blue) across a wide range of organisms, for mRNA (top) and pre-mRNA (bottom) molecules. Depletion is stronger, and observed in pre-mRNA as well. Grey indicates no data (zero IDS and zero TDS). Left: Same for nearly-perfect (>96% identity) structures.

**
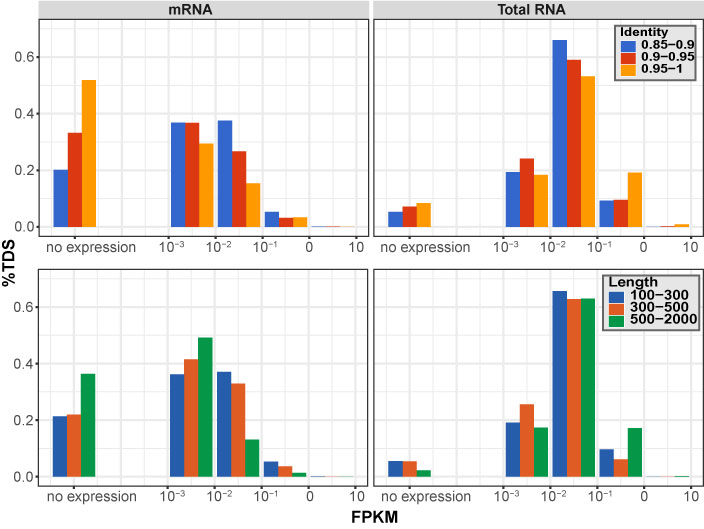
**

**Figure S3**: L**ong, nearly perfect, duplexes express more strongly in total-RNA.** The top two panels reproduce Fig. 4, panels A and B. For comparison, the bottom panels present the distribution of expression levels for the same regions in a total-RNA dataset. Expression is generally stronger, and the dependence on region’s size and identity disappear, supporting the notion that the low expression in mRNA samples is not due to technical reverse-transcription problems.

**
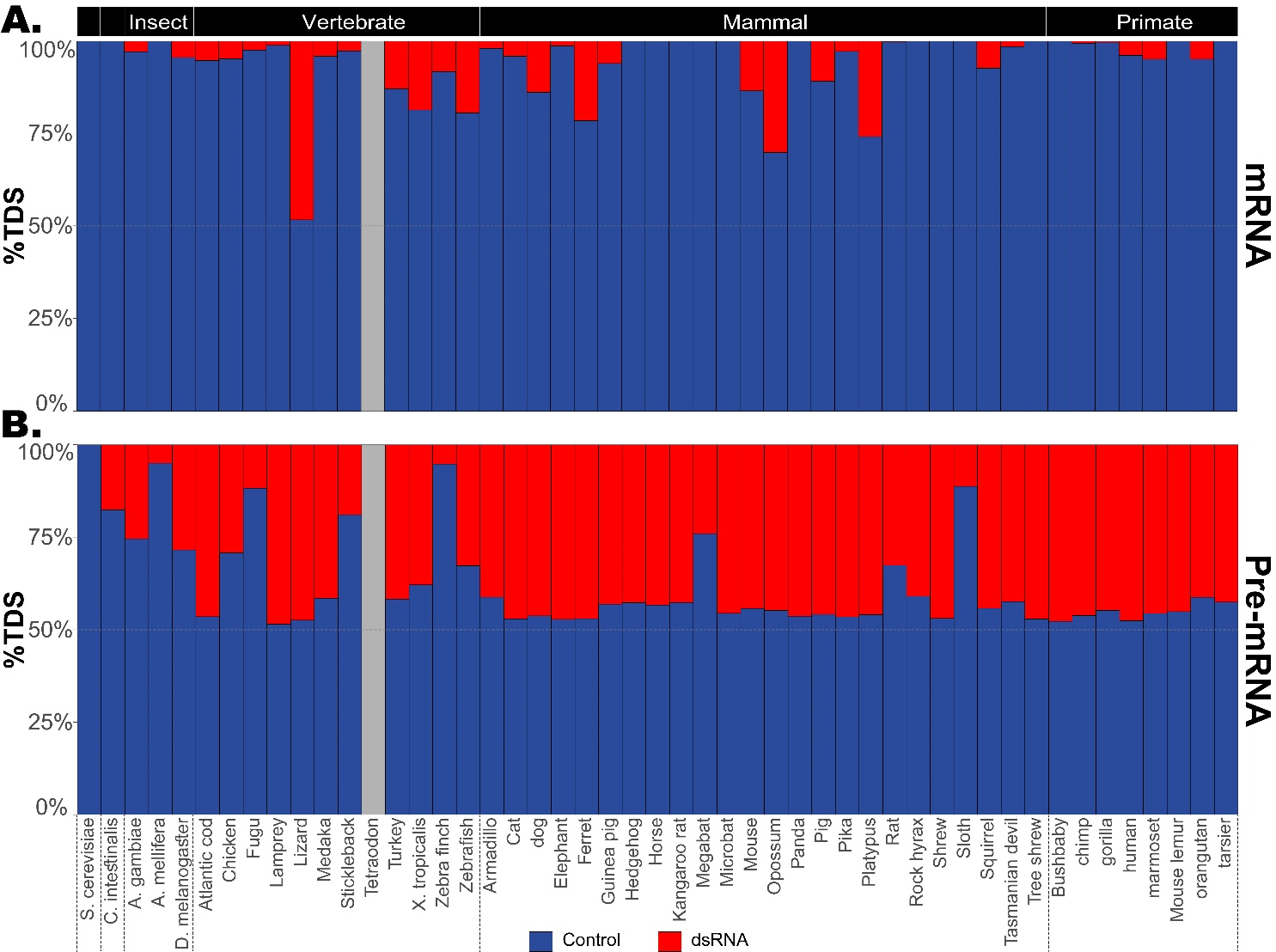
**

**Figure S4**: **Depletion of dsRNAs is stronger in non-repetitive mRNA regions**. Same as Figure 2, for non-repetitive regions.
