# Supplementary material for: Purifying selection of long dsRNA is the first line of defense against false activation of innate immunity
Source: Genome Biol. 2020 Feb 7;21:26. doi: 10.1186/s13059-020-1937-3 (PMC7006430; doi:10.1186/s13059-020-1937-3)
Supplement: Supplementary file 3 — Additional file 3: Review history. [file 13059_2020_1937_MOESM3_ESM.docx]

Review History

**First round of review**

**Reviewer 1**

**Are you able to assess all statistics in the manuscript, including the appropriateness of statistical tests used?** No, I do not feel adequately qualified to assess the statistics.

**Comments to author:**

This manuscript by Barak et al. is another ‘tour de force’ by the groups of Levanon and Eisenberg. In this manuscript, the authors describe how long dsRNA has been evolutionary cleaned from the genome of 49 organisms. The interrogated both genomic and transcriptome data and found that dsRNA was depleted from mRNA but had been present in pre-mRNA. They conclude that this is necessary to remove the dsRNA from mRNA as it would activate the innate immune receptors present in the cytoplasm. This is a very well planned and executed study.

I have one minor comments.

On page 7, sentence beginning on line 13 concerning genomic nucleotides should be rephrased, as it is difficult to understand.

**Reviewer 2**

**Are you able to assess all statistics in the manuscript, including the appropriateness of statistical tests used?** There are no statistics in the manuscript.

**Comments to author:**

In the submitted manuscript, the authors presented an intriguing observation that across many species, inverted duplicated sequences (IDSs) are depleted in mature mRNAs relative to tandem duplicated sequences (TDSs). Overall, I think that the work is reasonably well done and the concept is also novel. Hence, the manuscript may be considered for publication in Genome Biology. Below are some comments that I have for improving the authors' work.  

Major comments:

1) I am wondering how the authors decided on the various thresholds for what constitutes a long dsRNA. At the start, the authors defined long dsRNAs as being at least 40bp long (top of page 5). Subsequently, they wanted to be more stringent and selected 96% identitiy and 300bp as cutoffs.

But the important question is, what exactly are the structural requirements of the RNA substrate for MDA5 activation? From literature, MDA5 is believed to be strongly activated by long (>2000bp) dsRNAs. Nevertheless, it can also be activated by dsRNAs as short as around 100bp (PMID: 22160685 and 23142662). Hence, how did the authors' 40bp and 300bp criteria come about? Moreover, I think it is currently unclear how many mismatches MDA5 can tolerate in a dsRNA before it cannot be activated.

Can the authors systematically vary the length of the dsRNAs as well as the percentage identity and show how the results change? In the discussion, the authors wrote that "short (<300bp) and imperfect (identity<96%) dsRNAs are also purified from mRNAs", so it looks like they have already examined the parameters. It would useful to present these analysis results, instead of simply mentioning them at the end.

2) On a related note to the first point, it is not difficult to find (in human) long dsRNAs in 3'UTRs of protein-coding transcripts, many of which are well edited and annotated in databases like RADAR and REDIportal. Hence, intuitively, it seems odd that the authors only managed to identify only 7518 IDSs (> 40bp) in mRNAs for all 49 organisms examined (page 5). Can the authors compare their human IDSs (> 40bp) obtained from BLAST with the genes that are annotated in RADAR/ REDIportal, especially those that have many editing sites in the 3'UTR?

3) In Figure 2A (mRNAs), orang utan and the chimpanzee have relatively large percentages of dsRNAs (i.e. the red bars are comparatively big for the two animals). This is curious, considering that their genomes are almost identical with that of human. Why is there such a discrepancy between orang utan/ chimp and human (as well as the rest of the primates)?

4) Can the authors explain how they deal with a mobile element that has >2 copies of itself within the same gene? For example, a mobile element may have duplicated itself twice in the same gene, once in the same direction and once in the opposite direction. In the authors' BLAST procedure, this will yield one TDS and two IDSs. Now let's say splicing removes just that one copy in the opposite direction. The single removal will then be counted as two IDSs lost in the mature mRNA. Hence, in such cases, there appears to be double counting and will cause the depletion of IDSs to be stronger than it really is.

5) There are no statistics indicating whether the depletion observed in any of the scenarios presented is significant. Please test for significance.

Minor comments:

1) Introduction: The authors wrote that the main function of ADAR1 "is to introduce mismatches to the endogenous dsRNAs while still in the nucleus" - It will be good to specify that they are referring to p110, since p150 is mainly cytoplasmic.

2) Page 5: The authors found 7518 IDSs covering 924kbp as well as 43,219 TDSs covering 2.67Mbp in mRNAs. Can the authors also provide the corresponding numbers of IDSs and TDSs in pre-mRNAs?  

3) Figure 2: Can the authors label the y-axis to indicate what the percentages refer to?  

4) Why did the authors examine only 30 RNA-seq GTEx samples (originating from 15 different tissues) when there are thousands of samples available?

5) The authors wrote that the IDS "regions that show no expression in mRNA-seq data are actually well covered in total-RNA datasets". Can the authors illustrate this with one or two examples in the main figure? (Also, comparing mRNA-seq with total-RNA-seq does not really rule out artifacts due to secondary structures, since mRNAs and pre-mRNAs are expected to exhibit different secondary structures.)

6) Figure 4A-B: What are the y-axis labels?

7) There are some spelling and grammatical errors. Please proofread.

**Authors Response**

**Point-by-point responses to the reviewers’ comments:**

We thank you and the reviewers for the overall positive evaluation of our manuscript “Purifying selection of long dsRNA is the first line of defense against false activation of innate immunity”. The paper was revised addressing all comments and suggestions made by the reviewers, as detailed below. We hope you find the current version of the manuscript suitable for publication in Genome Biology.

Below is our point-by-point response to all comments raised by the reviewers.

**Reviewer #1**:
This manuscript by Barak et al. is another ‘tour de force’ by the groups of Levanon and Eisenberg. In this manuscript, the authors describe how long dsRNA has been evolutionary cleaned from the genome of 49 organisms. The interrogated both genomic and transcriptome data and found that dsRNA was depleted from mRNA but had been present in pre-mRNA. They conclude that this is necessary to remove the dsRNA from mRNA as it would activate the innate immune receptors present in the cytoplasm. This is a very well planned and executed study.

I have one minor comments.
On page 7, sentence beginning on line 13 concerning genomic nucleotides should be rephrased, as it is difficult to understand.

We thank the referee for the positive evaluation of our work. The sentence was modified to read: “In fact, we do see that most dsRNA structures reside in repetitive sequences (counting unique dsRNA genomic nucleotides overlapping known repeats: 531/786kbp in mRNAs and 707/1260Mbp in pre-mRNAs, for organisms with repetitive elements annotation; Supp. Table S7).”

Reviewer #2: In the submitted manuscript, the authors presented an intriguing observation that across many species, inverted duplicated sequences (IDSs) are depleted in mature mRNAs relative to tandem duplicated sequences (TDSs). Overall, I think that the work is reasonably well done and the concept is also novel. Hence, the manuscript may be considered for publication in Genome Biology. Below are some comments that I have for improving the authors' work. 

Major comments:

1) I am wondering how the authors decided on the various thresholds for what constitutes a long dsRNA. At the start, the authors defined long dsRNAs as being at least 40bp long (top of page 5). Subsequently, they wanted to be more stringent and selected 96% identity and 300bp as cutoffs.

But the important question is, what exactly are the structural requirements of the RNA substrate for MDA5 activation? From literature, MDA5 is believed to be strongly activated by long (>2000bp) dsRNAs. Nevertheless, it can also be activated by dsRNAs as short as around 100bp (PMID: 22160685 and 23142662). Hence, how did the authors' 40bp and 300bp criteria come about? Moreover, I think it is currently unclear how many mismatches MDA5 can tolerate in a dsRNA before it cannot be activated.

Can the authors systematically vary the length of the dsRNAs as well as the percentage identity and show how the results change? In the discussion, the authors wrote that "short (<300bp) and imperfect (identity<96%) dsRNAs are also purified from mRNAs", so it looks like they have already examined the parameters. It would useful to present these analysis results, instead of simply mentioning them at the end.

We thank the reviewer for this important comment. Indeed, we have chosen the length and identity cutoffs based on analysis of the results as a function of length and identity. The information requested by the referee appears in Fig. 3A, showing that for lengths above 300bp or identity greater than 96% the depletion of dsRNAs becomes pronounced even for pre-mRNAs (and becomes stronger for mRNAs). We added a citation to PMID 22160685.

2) On a related note to the first point, it is not difficult to find (in human) long dsRNAs in 3'UTRs of protein-coding transcripts, many of which are well edited and annotated in databases like RADAR and REDIportal. Hence, intuitively, it seems odd that the authors only managed to identify only 7518 IDSs (> 40bp) in mRNAs for all 49 organisms examined (page 5). Can the authors compare their human IDSs (> 40bp) obtained from BLAST with the genes that are annotated in RADAR/ REDIportal, especially those that have many editing sites in the 3'UTR?

Indeed, there are hundreds of human transcripts harboring inverted repeats in their 3’UTR (that is, both arms of the dsRNA are included in the mature transcript). LL Chen et al (EMBO J. 2008) have found 333 such cases. Consistently, we find 347 dsRNAs within human mRNAs (see Table S2).

This number is higher than the average number per species, which is 133 (6525/49). Two possible reasons for the higher number found in human are the abundance of Alu repeats on one hand, and better annotation of the 3’UTRs (leading to longer UTRs, harboring more repetitive elements) on the other hand.

Most editing in 3’UTRs and most sites documented in RADAR and REDItools result from pairing with intronic Alu elements. These structures are included in the pre-mRNA IDSs.

3) In Figure 2A (mRNAs), orangutan and the chimpanzee have relatively large percentages of dsRNAs (i.e. the red bars are comparatively big for the two animals). This is curious, considering that their genomes are almost identical with that of human. Why is there such a discrepancy between orangutan/ chimp and human (as well as the rest of the primates)?

We thank the referee very much for this important question. It has helped us to identify two technical errors that we have now fixed. The pre-mRNA files used previously for human and gorilla were truncated (due to a premature termination of the download), and the table that mapped chimpanzee and orangutan genes to the genome was partially corrupted. We have fixed these input files, re-ran our analysis, and updated the figures, tables and numbers in the paper accordingly.

These changes do not affect our results qualitatively, except for eliminating the artifact pointed out by the referee for chimpanzee and orangutan.

Again, thank you very much for picking up this irregularity in our data.

4) Can the authors explain how they deal with a mobile element that has >2 copies of itself within the same gene? For example, a mobile element may have duplicated itself twice in the same gene, once in the same direction and once in the opposite direction. In the authors' BLAST procedure, this will yield one TDS and two IDSs. Now let's say splicing removes just that one copy in the opposite direction. The single removal will then be counted as two IDSs lost in the mature mRNA. Hence, in such cases, there appears to be double counting and will cause the depletion of IDSs to be stronger than it really is.

In order to avoid double counting, we have reported (in addition to the number of possible structures) the number of unique nucleotides that are part of these structures. Thus, in the example above, nucleotides of the single inverted repeat will be counted once even though it may form has two IDS structures.

However, the reviewer correctly points out that in specific cases there may be an inherent preference towards IDS or TDS. We thank the reviewer very much for this important insight.

Indeed, in the case of three elements, two of which in one direction and the third in the opposite direction, there are two IDSs and only one TDS. This combination favors IDSs then. However, if all three elements are on the same strand, there are three TDSs and no IDS. Statistically (assuming uniform strand distribution and independence), one expects ¾ of the triplets to be of the first kind and ¼ to be of the second kind, leading to an overall equal expected number of IDSs and TDSs, 1.5 each.

Then, if splicing removes one of the elements (let's say, the downstream one), one is left with two elements. In half of the cases these would be on the same strand, and in the other half they will be inverted. All in all, we again have an equal expected number of IDSs and TDSs, 0.5 each.

More generally, assume there are N elements in a transcript, distributed uniformly and independently between the two strands. The probability to find $m$ positive strand elements is then binomial:

$P\left( m \right)=\left( \begin{matrix} N \\ m \end{matrix} \right)2^{-N}$

Assuming $m$ positive strand elements, the numbers of IDSs and TDSs within the transcript are:

$$\#TDS=m\left( m-1 \right)+(N-m)(N-m-1);\begin{matrix} & & \#IDS= \end{matrix}m(N-m)$$

The expected number of IDSs, averaged over all transcripts with N elements is then given by

$$\begin{matrix} \left\langle IDS \right\rangle& = & \sum_{m=0}^{N} P\left( m \right) m\left( N-m \right) \\ & = & \sum_{m=0}^{N} \left( \begin{matrix} N \\ m \end{matrix} \right)2^{-N} m\left( N-m \right) \\ & = & N(N-1)/4 \end{matrix}$$

which is exactly half of the possible pairs (It is easy to calculate in the same way the expected number of same strand pairs, $\left\langle TDS \right\rangle$, leading to the same result, as expected). That is, although for each certain configuration (specific value of $m$, the number of positive strand elements) the numbers of possible IDSs and TDSs may differ, the average over all values of $m$ restores the symmetry and yields equal number of possible IDSs and TDSs.

Although this is an important observation which we deeply appreciate, we feel that it is too technical to include in the paper, given that the bottom line confirms the naively expected symmetry.

5) There are no statistics indicating whether the depletion observed in any of the scenarios presented is significant. Please test for significance.
All comparisons presented are obviously highly significant, but we agree it is better to add actual p-values. For each comparison of IDSs and TDSs, we used the statistic #IDS/(#IDS+#TDS), the observed fraction of IDSs among all duplicated sequences found, and calculated the p-value using the null hypothesis that IDS and TDS are equally probable (i.e. a single proportion test with H0 being the fraction equals 0.5, up to finite sample fluctuations).

These p-values now appear throughout the text (in all cases but one, p<1e-100).

Minor comments:

1) Introduction: The authors wrote that the main function of ADAR1 "is to introduce mismatches to the endogenous dsRNAs while still in the nucleus" - It will be good to specify that they are referring to p110, since p150 is mainly cytoplasmic.

We have modified the text to read “It was pointed out recently that the main function of the essential ADAR1 enzyme, conferring Adenosine to Inosine (A-to-I) RNA-editing, is to prevent activation of cytosolic immune response [6-10]. A-to-I editing, mostly carried out by the constitutive ADAR1p110 variant, introduces mismatches to the endogenous dsRNAs while still in the nucleus [11-12] so that the edited endogenous transcripts are no longer recognized by viral dsRNA sensors in the cytoplasm.”

2) Page 5: The authors found 7518 IDSs covering 924kbp as well as 43,219 TDSs covering 2.67Mbp in mRNAs. Can the authors also provide the corresponding numbers of IDSs and TDSs in pre-mRNAs?

We have added these numbers to the main text (these and all other data are also included in Supp. Tables). The numbers are slightly different in the present version, mainly thanks to elimination of the technical error in chimpanzee and orangutan (see above). The amended sentence reads: “Furthermore, the selection against dsRNAs is much weaker in the pre-mRNA, suggesting it is driven by cytoplasmic processes (57.5M IDSs covering 1.26Gbp, compared with 61.3M TDSs covering 1.51Gbp; p<1e-100)”.

3) Figure 2: Can the authors label the y-axis to indicate what the percentages refer to?

We have added the label “%TDS”, to reflect that the blue bars represent the fraction of TDSs out of both types of structures.

4) Why did the authors examine only 30 RNA-seq GTEx samples (originating from 15 different tissues) when there are thousands of samples available?

Full calculation of the editing index including hyper-edited reads is highly time-consuming. The randomly chosen thirty samples provided sufficient reads’ coverage for the purposes of the present study.

5) The authors wrote that the IDS "regions that show no expression in mRNA-seq data are actually well covered in total-RNA datasets". Can the authors illustrate this with one or two examples in the main figure? (Also, comparing mRNA-seq with total-RNA-seq does not really rule out artifacts due to secondary structures, since mRNAs and pre-mRNAs are expected to exhibit different secondary structures.)

Figure 4 is quite busy as is, so we have a supplementary figure that compares the coverage distribution of these regions between the 15-tissues pool and a dataset of total RNA (Supp. Figure 3), showing that the expression level observed in total-RNA is higher, and the dependence on length and identity disappears in total RNA.

The referee is correct in saying the structures in mRNA and pre-mRNA are different. However, as almost all of the regions studied here reside in introns, their expression in mRNA reflects rare cases of intron retention, in the mRNA and pre-mRNA molecules are similar (at least in the immediate vicinity of the region).

6) Figure 4A-B: What are the y-axis labels?

These panels present distributions, and the we have added the label “Fraction”.

7) There are some spelling and grammatical errors. Please proofread.

Done.

**Second round of review**

**Reviewer 2**

I am satisfied with the authors' revisions. It is a good piece of work.
